# Supplementary material for: Building compositional tasks with shared neural subspaces
Source: Nature. 2025 Nov 26;650(8100):164–72. doi: 10.1038/s41586-025-09805-2 (PMC12872450; doi:10.1038/s41586-025-09805-2)
Supplement: Supplementary file 1 — Reporting Summary [file 41586_2025_9805_MOESM1_ESM.pdf]

nature portfolio

Corresponding author(s): *Double-anonymous peer review submissions: write DAPR and your manuscript number here instead of author names.*

Last updated by author(s): *YYYY-MM-DD*

Reporting Summary

Nature Portfolio wishes to improve the reproducibility of the work that we publish. This form provides structure for consistency and transparency in reporting. For further information on Nature Portfolio policies, see our Editorial Policies and the Editorial Policy Checklist.

Statistics

For all statistical analyses, confirm that the following items are present in the figure legend, table legend, main text, or Methods section.

n/a	Confirmed	
☐	☒	The exact sample size (*n*) for each experimental group/condition, given as a discrete number and unit of measurement
☐	☒	A statement on whether measurements were taken from distinct samples or whether the same sample was measured repeatedly
☐	☒	The statistical test(s) used AND whether they are one- or two-sided<br>*Only common tests should be described solely by name; describe more complex techniques in the Methods section.*
☐	☒	A description of all covariates tested
☐	☒	A description of any assumptions or corrections, such as tests of normality and adjustment for multiple comparisons
☐	☒	A full description of the statistical parameters including central tendency (e.g. means) or other basic estimates (e.g. regression coefficient) AND variation (e.g. standard deviation) or associated estimates of uncertainty (e.g. confidence intervals)
☐	☒	For null hypothesis testing, the test statistic (e.g. *F*, *t*, *r*) with confidence intervals, effect sizes, degrees of freedom and *P* value noted<br>*Give P values as exact values whenever suitable.*
☒	☐	For Bayesian analysis, information on the choice of priors and Markov chain Monte Carlo settings
☒	☐	For hierarchical and complex designs, identification of the appropriate level for tests and full reporting of outcomes
☐	☒	Estimates of effect sizes (e.g. Cohen's *d*, Pearson's *r*), indicating how they were calculated

Our web collection on statistics for biologists contains articles on many of the points above.

Software and code

Policy information about availability of computer code

Data collection	We presented stimuli and recorded behavioral responses using Psychtoolbox (version 3) and MATLAB (version 2015a). The behavioral training code can be provided upon request. Electrophysiological data was acquired using Plexon Omniplex recording system (Plexon Inc., Dallas, TX.) Eye tracking data was gathered with an EyeLink 1000 (SR Research, software version 5.09). Neuron waveforms were sorted using Plexon's Offline Sorter (version 4).
Data analysis	The data underwent analysis utilizing both built-in functions and custom code developed in MATLAB (version 2024b & version 2021b). Built-in functions include fitclinear for logistic regression, lassoglm for fitting GLM models, pca for dimensionality reduction, corr for Pearson's correlation, and fminsearch for fitting psychometric curves. These are highlighted in the corresponding methods section. To quantify the statistical significance of trends, we used Trend-Free PreWhitening (TFPW) as implemented in MK_tempAggr.m function (mannkendall Version 1.1.0 ,https://mannkendall.github.io/Matlab) and referenced in Coen et al. 2020. The stimuli were rendered as three-dimensional models using POV-Ray (version 3.7). To compute chi-squared test we used chi2test.m function implemented in MATLAB and referenced in Axensten P, chi2test (https://www.mathworks.com/matlabcentral/fileexchange/16177-chi2test), MATLAB Central File Exchange (2007). Procedures for non-standard statistical procedures are detailed in the methods. Code for custom functions are either referenced in the manuscript with a pointer to the original source or are included in an online public repository (https://github.com/buschman-lab/CompositionalTasks) and on Zenodo (https://doi.org/10.5281/zenodo.17274345).

For manuscripts utilizing custom algorithms or software that are central to the research but not yet described in published literature, software must be made available to editors and reviewers. We strongly encourage code deposition in a community repository (e.g. GitHub). See the Nature Portfolio guidelines for submitting code & software for further information.

Data

Policy information about availability of data

All manuscripts must include a data availability statement. This statement should provide the following information, where applicable:

- Accession codes, unique identifiers, or web links for publicly available datasets
- A description of any restrictions on data availability
- For clinical datasets or third party data, please ensure that the statement adheres to our policy

Processed data have been deposited on the FigShare archive and can be accessed at https://figshare.com/articles/dataset/Data_for_Tafazoli_et_al_2025_Building_Compositional_Tasks_with_Shared_Neural_Subspaces/30276238

Research involving human participants, their data, or biological material

Policy information about studies with human participants or human data. See also policy information about sex, gender (identity/presentation), and sexual orientation and race, ethnicity and racism.

Reporting on sex and gender	N/A
Reporting on race, ethnicity, or other socially relevant groupings	N/A
Population characteristics	N/A
Recruitment	N/A
Ethics oversight	N/A

Note that full information on the approval of the study protocol must also be provided in the manuscript.

Field-specific reporting

Please select the one below that is the best fit for your research. If you are not sure, read the appropriate sections before making your selection.

☒ Life sciences ☐ Behavioural & social sciences ☐ Ecological, evolutionary & environmental sciences

For a reference copy of the document with all sections, see nature.com/documents/nr-reporting-summary-flat.pdf

Life sciences study design

All studies must disclose on these points even when the disclosure is negative.

Sample size	As detailed in the manuscript, a total of 1,081 neurons were recorded from 5 brain regions across 2 animal subjects. The number of subjects (2) and the number of neurons recorded per region (64-480) follows previous work using similar approaches (e.g., Panichello and Buschman et al, 2021, Buschman and Miller, 2007; Mante et al, 2013; Siegel et al, 2015).
Data exclusions	To ensure statistical power, neurons were omitted from specific analyses if they did not meet a predetermined number of trials in any condition of interest. This exclusion criterion was predetermined and is delineated for each analysis in the methods section. Additionally, units that were only partially detected during recording sessions were excluded. All analyses presented in this manuscript were conducted on individual neurons, isolated based on their waveform.
Replication	Independent experiments were performed in 2 animals, with 1,081 neurons recorded across 34 days. All data was included (except for exclusions noted above). There were no failed replication attempts (i.e., no animals failed to learn the task). Neural recordings were excluded only if the animals did not perform predetermined number of blocks during a recording session.
Randomization	Each animal underwent exposure to every task manipulation. Task orders and trial conditions were randomized across trials within each session. Neurons were recorded without bias. Electrodes were positioned to optimize the signal-to-noise ratio of the electrophysiological signal without consideration of neural type or selectivity.
Blinding	Since all animals were allocated to a single experimental group, blinding was neither necessary nor feasible during behavioral training. Experimenters were blinded to experimental conditions while recording neurons and sorting waveforms into individual neurons.

Reporting for specific materials, systems and methods

We require information from authors about some types of materials, experimental systems and methods used in many studies. Here, indicate whether each material, system or method listed is relevant to your study. If you are not sure if a list item applies to your research, read the appropriate section before selecting a response.

Materials & experimental systems

n/a	Involved in the study	
☒	☐	Antibodies
☒	☐	Eukaryotic cell lines
☒	☐	Palaeontology and archaeology
☐	☒	Animals and other organisms
☒	☐	Clinical data
☒	☐	Dual use research of concern
☒	☐	Plants

Methods

n/a	Involved in the study	
☒	☐	ChIP-seq
☒	☐	Flow cytometry
☒	☐	MRI-based neuroimaging

Animals and other research organisms

Policy information about studies involving animals; ARRIVE guidelines recommended for reporting animal research, and Sex and Gender in Research

Laboratory animals	Two adult male rhesus macaques (Macaca mulatta) participated in the experiment. Monkeys Si and Ch were between 8 and 11 years old and weighed approximately 12.7 and 10.7 kg, respectively.
Wild animals	No wild animals were used in this study.
Reporting on sex	Both animals were male.
Field-collected samples	No field-collected samples were used in this study.
Ethics oversight	All experimental procedures were approved by the Princeton University Institutional Animal Care and Use Committee (protocol #3055) and were in accordance with the policies and procedures of the National Institutes of Health.

Note that full information on the approval of the study protocol must also be provided in the manuscript.

Plants

Seed stocks	N/A
Novel plant genotypes	N/A
Authentication	N/A
